# Deep Reinforcement Learning-Empowered Resource Allocation for Mobile Edge Computing in Cellular V2X Networks

**DOI:** 10.3390/s21020372

**Published:** 2021-01-07

**Authors:** Dongji Li, Shaoyi Xu, Pengyu Li

**Affiliations:** 1 School of Electronic and Information Engineering, Beijing Jiaotong University, Beijing 100044, China; 18111021@bjtu.edu.cn (D.L.); 16120090@bjtu.edu.cn (P.L.); 2 National Mobile Communications Research Laboratory, Southeast University, Nanjing 210096, China

**Keywords:** mobile edge computing, vehicle-to-everything, deep reinforcement learning, double deep q network

## Abstract

With the rapid development of vehicular networks, vehicle-to-everything (V2X) communications have huge number of tasks to be calculated, which brings challenges to the scarce network resources. Cloud servers can alleviate the terrible situation regarding the lack of computing abilities of vehicular user equipment (VUE), but the limited resources, the dynamic environment of vehicles, and the long distances between the cloud servers and VUE induce some potential issues, such as extra communication delay and energy consumption. Fortunately, mobile edge computing (MEC), a promising computing paradigm, can ameliorate the above problems by enhancing the computing abilities of VUE through allocating the computational resources to VUE. In this paper, we propose a joint optimization algorithm based on a deep reinforcement learning algorithm named the double deep Q network (double DQN) to minimize the cost constituted of energy consumption, the latency of computation, and communication with the proper policy. The proposed algorithm is more suitable for dynamic scenarios and requires low-latency vehicular scenarios in the real world. Compared with other reinforcement learning algorithms, the algorithm we proposed algorithm improve the performance in terms of convergence, defined cost, and speed by around 30%, 15%, and 17%.

## 1. Introduction

With the emergence of enormous numbers of intelligent devices, the mobile applications are blooming unbelievably, such as face recognition, natural language processing, augmented reality, autonomous driving, etc. [1]. Although mobile applications bring convenience and ease for users, the huge energy consumption and high latency of the computation exceed the capacity of users [2]. In particular, the aforementioned effects on vehicle-to-everything (V2X) communications are even more intolerable due to the high mobility and the constrained resources of the vehicles. The V2X communications scenario differs from other scenarios, such as text, voice, and video transmission. From Table 1, it is easy to observe that the low latency is the key part to guaranteeing the safety of passersby and passengers in V2X communications. In the IEEE 802.11p, namely, the present vehicular communication standards, the low latency is considered as a crucial criterion of V2X communications in 2009 [3]. Besides, since the global number of vehicles reached more than one billion in 2010 [4], the latency caused by the intensive loads or tasks of vehicular user equipment (VUE) is a bottleneck that needs to be broken through effectively. Moreover, with the increasing number of vehicles, the energy consumed for computation and transmission of the vehicles’ data exceeds our expectations continuously. Fortunately, the terrible situation is being alleviated with the aid of cloud computing.

As a promising technology, cloud computing has attracted significant attention in recent years for its multiple powerful resources, such as servers, storage devices, and network hardware [5]. As a result, cloud computing has become a vigorous and crucial paradigm of today’s communication service architecture. Cloud computing, which is viewed as the centralized data center commonly, provides enough computational resources for VUE that requires more computing abilities. Although cloud computing satisfies the more demands on computational resources, the long distance between the cloud server and VUE and the data transmission of the network will still cause some potential problems when the vehicles offload the huge volume of computing tasks to the cloud computing servers [6]. These issues hinder the achievement of low latency goals. Additionally, the energy consumption of the cloud servers and data centers contributes much to the total cost [7]. The cloud servers, as computing and data centers, may consume as much energy as 25,000 households. Furthermore, the energy costs of powering a typical data center double every five years [8]. A lot of efforts have been focused on how to explore more and more energy-efficient alternatives. Accordingly, mobile edge computing (MEC) is considered to reduce the energy consumed by cloud servers so that the more energy for the transmission can be saved.

Improving energy efficiency by closing the distance between distributed servers and VUE is a better solution than endlessly increasing the computing and storage resources for the centralized servers. MEC servers deployed at the cellular edge leverage their ability to provide the computational resources to reduce the latency caused by the long distance and the transmission of the tremendous data [9]. Specifically, the cloud servers could not meet the new demands of the Internet of Things (IoT) devices and applications. Moreover, the energy consumption of offloading tasks or data to MEC servers is less than that of the cloud servers for the shorter distance [10]. Motivated by the superiority of MEC, huge numbers of researchers are discussing the problem of how to utilize energy effectively with the help of the MEC computing paradigm widely. In light of the considerations about MEC, the European Telecommunications Standards Institute (ETSI) even formed the MEC Industry Specification Group (MEC ISG) standardization organization in late 2014 [11].

Unfortunately, the dynamic vehicular environment varies significantly and rapidly, so we cannot get the exact states of the environment. In particular, the states of vehicular environment rapidly and unpredictably change when vehicles move at high speed [12], which make it difficult for the conventional model-based methods to obtain reasonable and precise decisions. Furthermore, a few pieces of literature pay attention to the time-varying environment. For this unpredictable environment, model-free reinforcement learning algorithms are introduced to tackle the above problem [13,14,15]. In this paper, we study the total cost optimization constituted of energy consumption and latency caused by computation and communication. Nevertheless, this is non-deterministic polynomial hard (NP-hard). So far, NP-hard problems are still tough to solve by exact algorithms. Therefore, the most common approaches to solve them are approximation algorithms. We propose a joint optimization algorithm based on a deep reinforcement learning algorithm named the double deep Q network (double DQN). We deployed it on cloud servers i.e., a MEC controller, to make decisions and allocate all MEC servers’ resources in a centralized manner, rather than calculating the data offloaded from the VUE. Finally, the simulation results show that the joint optimization algorithm based on double DQN has a better performance compared with algorithms based on other reinforcement learning and deep reinforcement learning techniques. Overall, the main contributions in this paper can be summarized as follows.

- To solve the dynamic problem caused by the high speed of the vehicular environment, we propose a joint optimization algorithm based on double DQN, which comprehensively considers joint optimization, including offloading strategy, allocation of computational resources, and communication resources. Through building the neural networks to approximate the reward value of the whole system, our algorithm solves the joint optimization problem that traditional methods find hard to solve.
- We modify the reward function of double DQN and then use it iteratively to make the proper policy to minimize the long-term average cost so that the agent can obtain the correct guide in the interaction with the environment and gain a greater benefit from interactions.
- To fasten the training phase, we adopt offline training and on-ine implementation in this paper to accelerate the speed of the running and reduce the latency of computation. Besides, we also clean the redundant action space by discarding several inefficient actions for a faster training speed. The less time spent in the training phase, the faster the agent can react to adapt to the new vehicular environment. Therefore, vehicles can avoid potential risks.

The remainder of this paper is organized as follows. In Section 2 we review the related work and explain the motivations of this paper. A detailed introduction to the model system and problem formulation are described in Section 3. In Section 4, we introduce some brief background about deep reinforcement learning and present our proposed algorithm. Then the parameters, results, and analysis of simulations in this paper are represented in Section 5. In the final section, Section 6, we conclude the entire paper.

## 2. Related Work

Actually, a considerably large number of researchers and papers have paid a lot of attention to this field. The two main resources, the computation offloading and communication resources, need to be considered for optimization. To read easily and clearly, we classified the papers we refer to into two categories intuitively. In Table 2, we offer the summary comparison of the references based on some features, such as year, focus on computation offloading and communication resources, and the methods used. Computation offloading denotes the main topic of the current reference is the related work about computation offloading, such as matching the MEC servers and user equipment, for computation offloading decisions. Meanwhile, communication resources means the main topic of the reference is work about allocating wireless communication resources, such as selecting channels, assigning the transmission power, etc.

### 2.1. Computation Offloading

The first category considers how to generate or select a proper computation offloading strategy to minimize the different objectives—for instance, the defined cost, latency, or energy consumption. The authors in [16] studied the multi-user computation offloading problem for mobile edge cloud computing in a multi-channel wireless interference environment, and adopted one powerful mathematical tool, game theory, to achieve efficient computation offloading in a distributed manner. The authors of [17] also used one of the game theory approaches: a potential game to minimize the cost was constituted of energy consumption, latency, and even monetary cost. Some methods of convex optimization theory, such as the Lagrange method, were used to optimize their corresponding objectives, e.g., minimizing energy consumption in [10], reducing the consumption of the system consisted in [18], and enhancing the system computing capacity in [19]. Additionally, Alahmadi et al. proposed an interesting vehicular cloud network architecture where a group of vehicles near a traffic light cluster and form a temporal vehicular cloud by aggregating their computational resources in that cluster to minimize the processing and network power consumed in the data center of a cloud operator in [5]. Besides, aiming to achieve a trade-off between minimizing task completion time and data exchange costs, the authors modeled an undirected weighted graph between vehicular clouds and tasks. Although the above references optimized the corresponding computation offloading decisions in their papers, the communication resources were not taken into account. The licensed spectrum is limited, so it is impractical to ignore the optimization of the communication resources.

### 2.2. Jointly Optimizing the Computation Offloading and Communication Resources

The second category considers optimizing the computation offloading and the communications resources jointly to minimize the corresponding objectives. Zhao et al. studied how to jointly optimize the computation offloading strategy and the computational resources allocation strategy to minimize the system cost in [6]. Other than the conventional optimization methods, the methods of the game theory, such as the coalition game, the potential game, and the bargaining game, were used to optimize the corresponding objects in [20]. Zhang et al. developed a joint cloud and wireless resource allocation algorithm based on an evolutionary game considering mobile terminals’ energy consumption and time delay, and monetary cost in a mobile edge computing environment in [21]. Although the papers of the second category consider optimizing the computation offloading and communication resources simultaneously, it is still difficult to solve the unpredictable and dynamic vehicular environment caused by the high speed of the vehicles. However, the dynamic vehicular environment cannot be neglected.

Several studies utilized deep reinforcement learning in vehicular ad hoc networks to optimize their objects. Although their scenarios differ from that in this paper, the methods in their papers could help us to build the neural networks. Qi et al. provided a optimal offloading policy that mainly considers the future data dependency of the following tasks with the help of deep reinforcement learning algorithms in vehicular ad hoc networks in [22]. The authors of [24] used deep spatio-temporal residual networks with a permutation operator to predict the network traffic in order to reduce the capital expense and operating expense costs of commercial 5G-V2X networks. The authors of [25] adopted Q-learning to allocate transmission power, subchannels, and computing resources in a software-defined networking assisted MEC network architecture for the vehicular network.

## 3. Model System and Problem Formulation

**Figure 1:**
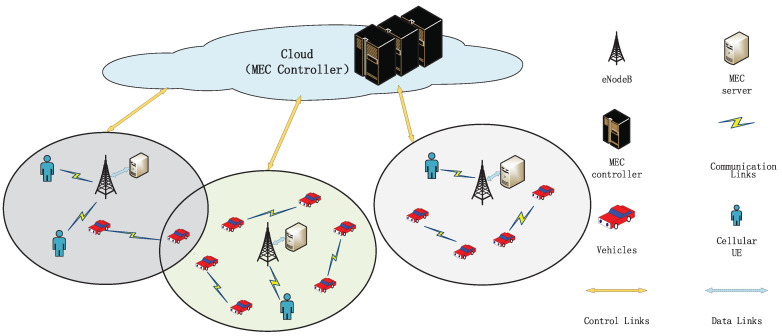
System model of the multi-cell scenario with multiple mobile edge computing (MEC) servers.

In this paper, as shown in Figure 1, we consider a MEC-enabled V2X system allowing VUE to offload their computing tasks to the MEC servers. This system operates in slotted time t∈{0,1,2,…}. Let $M=\left\{1,2,\ldots ,M\right\}$ denote the set of MEC servers (i.e., there are *M* base stations, since each base station is equipped with only one MEC server). The sets of VUE and cellular users are represented as $V=\left\{1,2,\ldots ,V\right\}$ and $N=\left\{1,2,\ldots ,N\right\}$, respectively. We consider an orthogonal frequency division multiple access (OFDMA) system for the cellular network, while VUE can reuse the identical resources. Thus, there is an interference between the cellular users and the VUE. Besides, there is the interference from the adjacent cells with the multi-cell scenario assumption in this paper.

### 3.1. Task Model

We assume that each VUE could generate one indivisible task to compute at one slot; i.e., the VUE only has two choices, offloading it to MEC to compute or computing locally. We employ a three-tuple $D_{v}=\left(d_{v},c_{v},T_{v}^{\max}\right)$ to express the VUE *v*’s task, where $d_{v}$ denotes the data size of the task, $c_{v}$ means the computational resources that VUE needs, and $T_{v}^{max}$ stands for the maximum delay that the system could endure. $\alpha_{v,m}\in \left\{0,1\right\}$ is used to indicate the offloading decision; $\alpha_{v,m}=0$ represents the VUE computes task locally; otherwise, VUE offloads task to one MEC server to compute. Furthermore, we define $\theta =\;\left\{\alpha_{v,m}|v\in V,m\in M\right\}$ as the decision variable and $X=\;\left\{\alpha_{v,m}\in \theta |\alpha_{v,m}=1\right\}$ as the offloading decision.

### 3.2. Local Computing Model

The local computing model is chosen as $\alpha_{v,m}=0$. At this time, task $D_{v}$ is computed by the local VUE, whose CPU frequency is expressed as $f_{v}^{l}$. The local computing delay can be formulated as

(1) $$T_{v}^{l}=\sum_{m=1}^{M}\frac{\left(1-\alpha_{v,m}\right)c_{v}}{f_{v}^{l}},\;\forall v\in V.$$

For a piece of VUE, the energy that is consumed by local computing is proportional to the coefficient of energy effect κ, and can be given as [26]

(2) $$E_{v}^{l}=\sum_{m=1}^{M}\left(1-\alpha_{v,m}\right)\kappa {\left(f_{v}^{l}\right)}^{2}c_{v}.$$

According to (1) and (2), the total overhead, i.e., total cost, of local computing model on VUE *v*, in terms of computational time energy, $C_{v}^{l}$ can be calculated as [26]

(3) $$C_{v}^{l}=\beta_{1}^{l}T_{v}^{l}+\beta_{2}^{l}E_{v}^{l},$$

where $0\leq \beta_{1}^{l},\beta_{2}^{l}\leq 1$ represent computational time and energy of VUE *v*, respectively.

### 3.3. Task Offloading Model

The task offloading model includes three phases, the task transmitting phase, the task computing phase, and the task return phase.

#### 3.3.1. Task Transmitting Phase

As aforementioned, VUE reuse the identical resources with cellular users in a multi-cell scenario. Consequently, not only the interference from cellular users who share the same spectra in the local cell, but also the interference from cellular users who reuse the identical resources in the adjacent cells is considered in this paper. The transmission power of VUE is defined as $P=\;\left\{p_{v}|0\leq p_{v}\leq P_{\max},\forall v\in V\right\}$, where $P_{\max}$ is the maximum transmission power. Then we derive the rate $R_{v}$ according to Shannon theory

(4) $$R_{v}=B\log\left(1+\frac{p_{v}h_{v}}{\sum_{i=1,i\neq v}^{V}\rho_{i}p_{i}h_{i}+I+\sigma^{2}}\right),$$

where $p_{v}$, $p_{i}$ represent the transmission power of VUE *v* and that of cellular user *i*, respectively. $h_{v}$ is the channel gain from VUE *v* to MEC servers, and $\rho_{i}$ is an indication variable. Notice that $\rho_{i}=1$ when VUE *v* reuses the resources of cellular user *i*, otherwise, $\rho_{i}=0$. $\sigma^{2}$ is the power of noise. In addition, I denotes the interference of devices located in the adjacent cell using the identical resources. This inter-cell interference is calculated as

(5) $$I=\sum_{u\in \psi }p_{u}h_{u},$$

where ψ denotes the set of VUE that shares resources with VUE *v*. $p_{u}$ is defined as the transmission power of VUE *u* that associates with the adjacent cell. In this paper, OFDMA is applied for a multi-cell scenario; hence, we consider the inter-cell interference caused by only one set of VUE in the adjacent cell. $h_{u}$ means the channel gain from VUE *v* to VUE *u*.

The Rayleigh fading channel model is utilized in this paper. Due to the high mobility of vehicles, the distance between senders and receivers rapidly changes, so we modify the channel gain as the following equation [27]

(6) $${\left|h_{i,j}\right|}^{2}=G\cdot {\left|d_{i,j}+v_{i,j}\cdot t_{w}\right|}^{\alpha }\cdot {\left|h_{0}\right|}^{2},$$

where *G* denotes the antenna gain, $d_{i,j}$ is the distance from sender *i* to receiver *j*, $v_{i,j}$ is the relative velocity of sender *i* to receiver *j*. $t_{w}$ is the waiting interval between the time point when the data are ready to transmit and the time point when the data start to be transmitted, which is assumed to be a constant in this paper. $h_{0}$ is the gain of Rayleigh fading channel.

According to (4) and (6), we derive the delay cost of task transmission as

(7) $$T_{v}^{t}=\sum_{m=1}^{M}\frac{\alpha_{v,m}d_{v}}{R_{v}}\;\forall v\in V,$$

#### 3.3.2. Task Computing Phase and Task Return Phase

In the task computing phase, the MEC servers will compute the VUE’s tasks, and then return the results to the corresponding users. The allocation policy of MEC server *m* is defined as $F=\;\left\{f_{m,v}|m\in M,v\in V\right\}$, where $f_{m,v}>0$ represents the computational resources that are allocated to VUE *v* by the MEC server *m*. Hence, the computing delay of the task that is offloaded from VUE *v* to MEC server *m* is given by

(8) $$T_{v}^{e}=\sum_{m=1}^{M}\frac{\alpha_{v,m}c_{v}}{f_{m,v}},\;\forall v\in V.$$

Compared with the delay of the task offloading phase, that of the task return phase is trivial due to the large rate of the downlink channel, so we ignore the delay of the task return phase.

Then we derive the sum delay cost and the sum energy cost according to (7) and (8), respectively

(9) $$T_{v}^{u}=T_{v}^{t}+T_{v}^{e}$$

(10) $$E_{v}^{u}=p_{v}T_{v}^{t}.$$

The sum cost of VUE *v* in the task offloading model in regard to time spend and energy consumed is computed as [28]

(11) $$C_{v}^{u}=\beta_{1}^{u}T_{u}^{e}+\beta_{2}^{u}E_{v}^{u},$$

where $0\leq \beta_{1}^{u},\beta_{2}^{u}\leq 1$ represent the sum delay and the energy consumed by transmission, respectively.

Finally, we define $C_{sum}$ to denote the sum cost of all VUE in local computing model and task offloading model, meanwhile give the equation of $C_{sum}$ as follows:

(12) $$C_{sum}=\sum_{v=1}^{V}\left(C_{v}^{l}+C_{v}^{u}\right).$$

### 3.4. Problem Formulation

In this paper, we optimize jointly the task offloading decision *X* and the transmission power policy *P* of VUE, along with the computational resources allocation policy *F* of MEC servers. The optimization problem is defined to minimize the sum cost of all VUE, and then given by

(13) $$\begin{array}{ccc} \min_{\left\{X,P,F\right\}} & C_{sum} & \\ s.t. & & \\ C1: & \alpha_{v,m}\in \left\{0,1\right\} & \forall v\in V,m\in M\\ C2: & \sum_{m\in M}\alpha_{v,m}\leq 1 & \forall v\in V\\ C3: & T_{v}^{l}+T_{v}^{u}\leq T_{v}^{\max} & \forall v\in V\\ C4: & p_{v}\leq P_{\max} & \forall v\in V\\ C5: & f_{m,v}>0 & v\in V,m\in M\\ C6: & \sum_{i=1}^{V}f_{v,m}\leq f_{m} & \forall m\in M\\ C7: & f_{m}\leq F_{m} & \forall m\in M \end{array}$$

C1 represents the offloading decision of VUE. C2 demands that VUE *v* just chooses at most one MEC server to offload its task. C3 indicates the constraint of the entire procedure delay. C4 is the constraint of the maximum transmission power of VUE *v*. C5 ensures every VUE could obtain several computational resources of MEC servers. C6 requires that the allocating computational resources should not exceed the remnant computational resources of MEC server *m*. C7 presents that the remnant computational resources should be less than the maximum computational resources that MEC servers can provide.

There are many ways to solve the (13), which is a mixed integer nonlinear programming problem, such as dividing this origin problem into several subproblems, and then obtaining the sub-optimal solution with the help of the optimal solution of these subproblems, or getting the sub-optimal solution of the origin problem via some heuristic algorithms. However, the aforementioned methods have better performance in the statistic scenario, which is contrary to the dynamic scenario of this paper.

Due to the mobility, the unpredictable states of vehicles lead to imprecise information of states at different time intervals. Although the optimal solution could be calculated by conventional optimal methods, these methods consume more intolerable time and space. To tackle this problem, we propose an approach based on double DQN, which is viewed as the evolution of deep Q networks, to solve the origin problem (13) directly in this paper. The proposed approach based on deep reinforcement learning will be detailed in the following Section 4.

## 4. Joint Optimization Based on Deep Reinforcement Learning

In this section, we propose a joint optimization algorithm based on deep reinforcement learning to optimize the aforementioned problem. Firstly, we give a brief introduction of the background on deep reinforcement learning.

### 4.1. Background on Double Deep Q Networks

#### 4.1.1. Q Learning

As an effective tool with which to solve complex issues, especially the model-free based problems, reinforcement learning has been said to be an important part of machine learning. By leveraging reinforcement learning, it is convenient to obtain satisfactory performance. As a famous and classical reinforcement learning algorithm, deep Q networks were proposed by *Watkins* in 1992 to find a simple way for agents to learn how to act optimally in controlled Markovian domains [13]. For a policy π, define Q values or action-values under state *s* executing action *a* as

(14) $$Q^{\pi }\left(x,a\right)=R_{x}\left(a\right)+\gamma \sum_{y}P_{xy}\left[\pi \left(x\right)\right]V^{\pi }\left(y\right),$$

where $R_{x}\left(a\right)$ denotes the immediate reward under state *s* executing action *a*, $\gamma \in \left(0,1\right)$ is the discount factor. With the increase of the iterative time in (14), the value of the second half of (14), which means how much the proportion of the future reward dominated in the whole decision procedure, will also increase exponentially. $P_{xy}\left[\pi \left(x\right)\right]$ means the transfer probability from state *y* to state *x* through action policy π[x]. The Equation (14) also can be explained intuitively as that the agent expects to obtain the immediate reward $R_{x}\left(a\right)$ via executing policy π[x], and then it moves to the state *x*, which is worthy $V^{\pi }\left(y\right)$ to it, with probability $P_{xy}\left[\pi \left(x\right)\right]$.

However, (14) is not suitable for model-free problems because of the existence of transfer probability. Fortunately, taking the Markovian property into consideration, the temporal differences (TD) method that learns from the continuous environments step by step was proposed by *R. Sutton* in 1988 [14]. The Q-learning iterative equation for model-free problems is given by

(15) $$\begin{array}{cc} Q_{\pi }\left(s,a\right)\; & =Q_{\pi }\left(s,a\right)+\\ \; & \alpha \left(R_{x}\left(a\right)+\gamma \max_{a^{\prime }}Q_{\pi^{*}}\left(s^{\prime },a^{\prime }\right)-Q_{\pi }\left(s,a\right)\right), \end{array}$$

where $\alpha =\frac{1}{N(s)}\in \left[0,1\right]$ is a discounted factor. N(s) originates from Monte-Carlo (MC) methods and represents the number of the iterative times start from the original state $s_{0}$ to the current state *s*.

#### 4.1.2. Deep Q Networks

**Figure 2:**
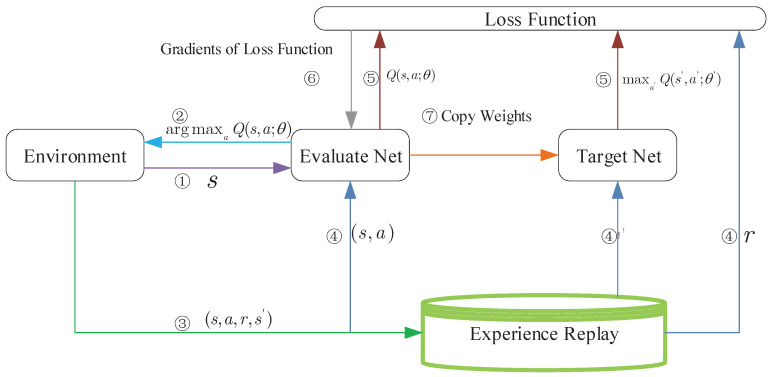
DQN algorithm frame diagram.

Although reinforcement learning has an anticipated effect, with the rapid extension of state space of agents, the Q table increases sharply so that it will spend a lot of time to search the policy corresponding to the maximum Q value, which is called the curse of dimensionality. To this end, the *Google DeepMind* group proposed a novel approach named deep Q networks, which replaces the Q table with approximation by neural networks [29]. The DQN algorithm frame diagram is shown as Figure 2. The crucial ideas behind the DQN algorithm are (1) to approximate the value function via neural networks to avoid the curse of dimensionality; (2) target net produces labels to train the evaluate net directionally; (3) randomly pick up experiences from experience replay to train in order to break the correlation among them. Loss function always is regarded as a significant part of deep reinforcement learning algorithms, because it builds a bridge over the gap between the evaluate net and the target net. The loss function of DQN is defined as

(16) $$\begin{array}{cc} {\mathrm{Loss}}^{DQN}= & \left(r+\gamma Q\left(s,{\mathrm{argmax}}_{a}Q\left(s^{\prime },\cdot ,\theta^{\prime }\right),\theta^{\prime }\right)\right.\\ \; & -Q(s,a,\theta )), \end{array}$$

where four-tuple $\left(s,a,r,s^{\prime }\right)$ represents experience that is sampled randomly from the experience reply, θ and $\theta^{\prime }$ are the weights of the evaluate net and target net respectively.

#### 4.1.3. Double Deep Q Networks

**Figure 3:**
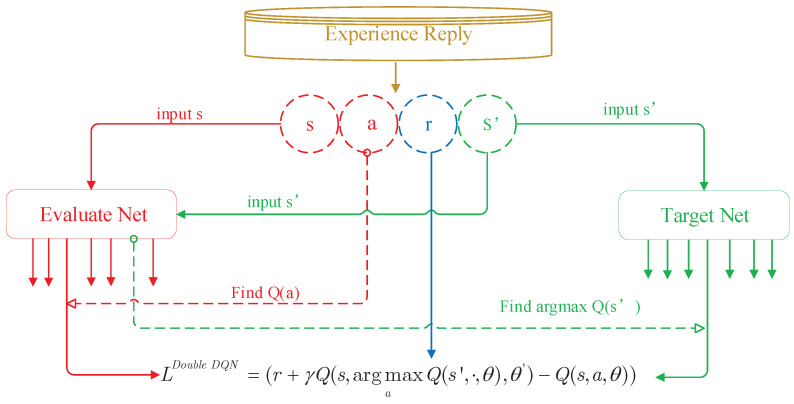
Double DQN algorithm frame diagram.

Even though the outperformance in solving model-free problems, DQN is still castigated by the overestimate owing to the insufficiently flexible function approximation and noise [15]. In particular, the performance of DQN algorithm will get worse when the overestimate does not occur uniformly [30]. For addressing this problem, *Google DeepMind* group utilized the idea behind [31] that proposed by *Hasselt* in 2010 to modify the DQN algorithm and proposed double DQN, which is envisioned as the evolution of DQN algorithm [32]. As shown in Figure 3, the major difference between DQN algorithm and double DQN algorithm is the loss function. The loss function of double DQN is given by

(17) $$\begin{array}{cc} {\mathrm{Loss}}^{DoubleDQN}= & (r+\gamma Q\left(s,{\mathrm{argmax}}_{a}Q\left(s^{{}^{\prime }},\cdot ,\theta \right),\theta^{{}^{\prime }}\right)\\ \; & -Q(s,a,\theta )). \end{array}$$

Then the evaluate net is updated by adopting mini-batch gradient descent (MBGD) in every step and copies its weights to target net every several steps. The gradient is written as

(18) $$\begin{array}{cc} \nabla_{\theta }Loss^{DoubleDQN}= & (r+\gamma Q\left(s,\underset{a}{\mathrm{argmax}}Q\left(s^{\prime },\cdot ,\theta \right),\theta^{\prime }\right).\\ \; & -Q\left(s,a,\theta \right))\nabla_{\theta }Q\left(s,a,\theta \right), \end{array}$$

where $\nabla_{\theta }f\left(\cdot \right)$ represents the gradient vector of f(·) with respect to θ.

### 4.2. Joint Optimization Algorithm Based on Double DQN

In this paper, we jointly optimize the task offloading decision vector *X*, the transmission power policy *P* of VUE, and the computational resources allocation policy *F* of MEC servers with the help of double DQN algorithm. Then the MEC controller deployed in the cloud is viewed as the agent, which could make the decision and coordinate the communications resources and the computational resources of all MEC servers.

To elaborate our proposed algorithm clearly and conveniently, several definitions about Markov decision process are defined below.

#### 4.2.1. State

At the beginning of every slot, the MEC servers will add their remnant computational resources as one part of the state. As the size of every task is time-varying, thus the remnant computational resources are different. Furthermore, the computational resources of the current slot only correlate with that of the prior slot, which satisfies the Markovian property. The definition of state space is written as

(19) $$S\left(t\right)=\{k_{i}\left(t\right),c_{v}\left(t\right),d_{v}\left(t\right)\}\;\forall v\in N,\forall i\in M,$$

where S(t) denotes the state space at slot *t*, and $k_{i}\left(t\right)$ is the remnant computational resources of MEC server *i* at slot *t*. The $c_{v}\left(t\right)$ and $d_{v}\left(t\right)$ indicate the size of a task and the necessary computational resources of VUE *v*.

#### 4.2.2. Action

To avoid the huge action space caused by the continuous action, we make the action discretization. For the task of VUE in this paper, there are three models, i.e., local computing, offloading task to the local MEC server, and offloading task to the adjacent MEC server could be chosen. Then we define the action vector A as

(20) $$A\left(t\right)=\left\{X,f_{1},\cdots ,f_{i},\cdots ,f_{N},p_{1},\cdots ,p_{i},\cdots ,p_{N}\right\},$$

where *X* represents the offloading decision vector of the VUE, $f_{i}$ means the computational resources that the MEC server allocates to the VUE *i*, and $p_{i}$ denotes the transmission power of VUE *i*. Notice that all the above action variables are decided centralizedly by the MEC controller.

#### 4.2.3. Reward

For the agent, the reward plays a primary role in guiding the interaction between the agent and environment. Consequently, we modify the immediate reward according to the conventional reward construction and rewrite an appropriate reward equation below

(21) $$R\left(s,a\right)=\frac{C_{local}-C_{sum}\left(s,a\right)}{C_{local}},$$

where $C_{local}$ means the sum local cost of all VUE. The definition of $C_{local}$ is shown as

(22) $$\sum_{v\in V}C_{v}^{l}=\sum_{v\in V}\left(\beta_{1}^{l}T_{v}^{l}+\beta_{2}^{l}E_{v}^{l}\right).$$

Combining the TD methods of (15) with the immediate reward of (21), we rewrite the original object (13) as

(23) $$\begin{array}{cc} Q_{\pi }\left(s,a\right) & =E_{\pi }\left[\sum_{t=1}^{T}\gamma^{t-1}R\left(t\right)\right]\\ \; & =E_{\pi }\left[\sum_{t=1}^{T}\gamma^{t-1}\left(\frac{C_{local}\left(t\right)-C_{sum}\left(t\right)}{C_{local}\left(t\right)}\right)\right]. \end{array}$$

Then modify the above object with the help of *Bellman Equation* as following

(24) $$\begin{array}{cc} Q_{\pi }\left(s,a\right)= & \left(1-\alpha \right)Q_{\pi }\left(s,a\right)+\alpha (R\left(s,a\right)\\ \; & +\gamma \max_{a^{\prime }}Q_{\pi }\left(s^{\prime },a^{\prime }\right)). \end{array}$$

For MEC controller, the destination of interacting with the environment and learning from experience is to find the optimal policy to maximize the long-term discounted sum reward, i.e., maximize the Equation (24). Then the optimial policy can be expressed below

(25) $$\pi^{*}=\arg\max_{a\in A}Q_{\pi }\left(s,a\right).$$

To explore the remainder of the action space sufficiently, the ϵ-greedy algorithm is utilized in this paper, which could achieve the trade-off between the exploitation and the exploration with the probability ϵ. Although the discretization of action is used in this paper, the action space is still enormous as the result of the increase of action’s dimension. Accordingly, we remove several inefficient policies to accelerate the speed of the training phase and alleviate the latency caused by these inefficient policy. For instance, the inefficient allocation policies will be abandoned, because these inefficient policy allocate the computational resources for VUE to exceed the maximum remnant computational resources of MEC servers.

Furthermore, since the high mobility of vehicles, the current cell that VUE associate might alter at next slots, which will trigger the exponential extension of the the action space with the increase of VUE quantity. Consequentially, we make the preprocess for the action space before the MEC controller decides the policy, e.g., the VUE will choose the local computing model firstly when the delay of local computing $T_{v}^{l}$ is less than the maximum delay that the system can endure $T_{v}^{max}$. With the aforementioned preprocess for action space, the train speed and the size of action space can be improved significantly.

### 4.3. Four Phases of Joint Optimization Algorithm

**Figure 4:**
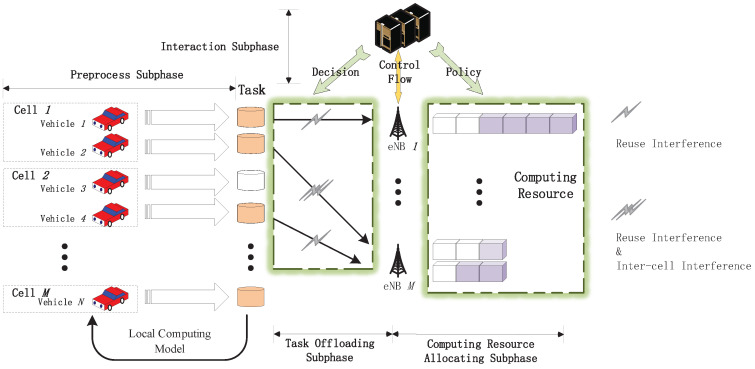
Four subphases diagram.

Since the whole procedure of we proposed algorithm is too long to be read clearly, for convenience and concise representation of the process, we separate the entire procedure into four subphases, as shown in Figure 4.

#### 4.3.1. Preprocess Subphase

As mentioned above, the VUE produce at most one task every slot, then they will judge whether they need the help from MEC servers. If the delay of local computing is less than the maximum delay that they can endure, i.e., $T_{v}^{l}\leq T_{v}^{max}$, they will choose the local computing model. Notice that the offloading policies of their task are regarded as inefficient actions at this time, i.e., the MEC controller will not consider offloading the task of these VUE to MEC servers. Simultaneously, other VUE report the information about the data size of task $d_{v}$ and the computational resources $c_{v}$ that these VUE require to the local MEC server.

#### 4.3.2. Interaction Subphase

After receiving the information about the task of VUE, the MEC servers combine this information and the information about their remnant computational resources together. Then the MEC servers send combined information to the MEC controller immediately. As the centralized decision-maker, MEC controller will discard the allocation policies, which allocate the computational resources for VUE to exceed the maximum computational resources of MEC servers. Next, the MEC controller concatenates the reported information as the state and input it into neural networks. Then, according to our proposed algorithm, the neural networks will output an appropriate policy including the offloading decision *X*, the transmission power policy *P*, and the computational resources allocation policy *F*. At the end of this subphase, all MEC servers receive the policy from the MEC controller and broadcast it to the VUE in the current cell. For reducing the latency of interaction subphase, we train the double DQN offline, just deploy it online.

#### 4.3.3. Task Offloading Subphase

With the instruction of the MEC servers, the VUE offload the task to the assigned MEC server with the corresponding transmission power. As displayed in Figure 4, the single gray flash symbol indicates that there is interference produced by VUE reusing the identical radio resources with cellular users. Apart from the single flash symbol, the double gray flash symbols represent the coexistence of the interference produced by reusing the same radio resources and the interference produced by the user, who is in the adjacent cell and reuse the identical communication resources with this VUE coexist.

#### 4.3.4. Computational Resources Allocating Subphase

In the computational resources allocating subphase, the MEC servers will allocate the suitable computational resources to the corresponding tasks according to the computational resources allocation policy *F*. Although the initial computational resources of every MEC servers is identical, the remnant computational resources is different for the varied size of tasks.

## 5. Simulations and Analyses

In this section, the simulation results of our proposed joint optimization algorithm based on double DQN (JOADD) are presented in comparison to two algorithms based on other reinforcement learning or deep reinforcement learning, which are named the joint optimization algorithm based on DQN (JOAD) and the joint optimization algorithm based on Q-learning (JOAQ). The simulations are based on the Keras program in Python form. Keras is a deep learning API written in Python, running on top of the machine learning platform TensorFlow. The version of Python used in our work was 3.6.

As aforementioned, a multi-cell scenario with multiple pieces of VUE is considered in this paper. Assume that M=3 MEC servers are available to provide services for VUE. Each MEC server has varying maximum computational resources which range from 1 to 6 GHz/s in different scenarios. The total number of pieces of VUE is also varying, whereas each VUE’s local computational resources are constant, namely, $f_{v}=1$ GHz/s. The size of VUE’s task $d_{v}$ is assumed as a discrete value that is sampled uniformly from [500, 800] kbits. Meanwhile, the number of CPU cycles $c_{v}$ that the task requires is proportional to the size of the task. The detailed values of all above parameters are listed in Table 3.

Moreover, the architecture of neural networks is also a crucial part of fitting the core of the algorithm, namely, the Q table. However, there is no theoretical and mathematical support to guide one through how to build the best neural networks. Consequently, we built the double DQN with the help of our experience. The double DQN consists of four layers, including one *inputs* layer, two *dense* layers as the hidden layers, and one *dense* layer as the outputs layer. The number of neurons of the inputs layer and that of the outputs layer are identical; the numbers are equal to the dimension of the state space and that of the action space respectively. The other parameters about neural networks are listed in Table 4.

**Figure 5:**
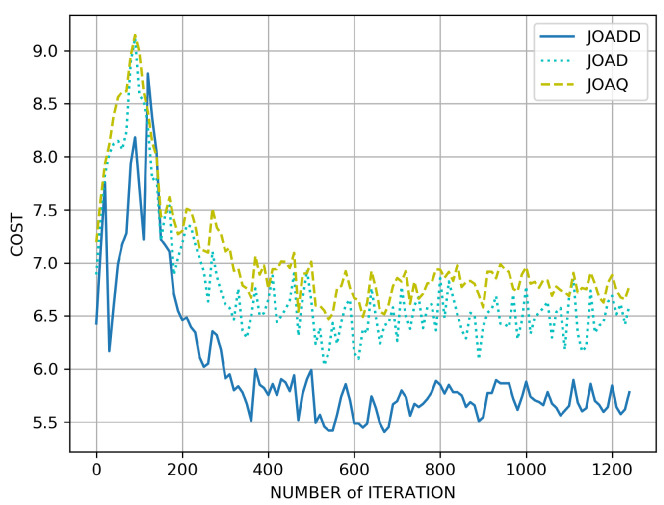
The convergence of different algorithms.

We present the convergences of three algorithms under the same conditions in Figure 5. As shown, after about 300 iterations, the fluctuation of JOAQ begins to decline first. Then about 400 iterations, the JOADD and JOAD start to flatten their fluctuations. The reason for the above phenomenon is that the Q-learning turns the continuous states into discrete ones to reduce the size of state space to avoid the curse of dimensionality. For the same reason, JOAQ might miss a better policy due to the discretization of the space. However, JOAD and JOADD utilize the neural networks to approximate the discrete Q-table; thus, above algorithms have better performance than JOAQ. Furthermore, in the light of eliminating the overestimate, JOADD has better performance.

**Figure 6:**
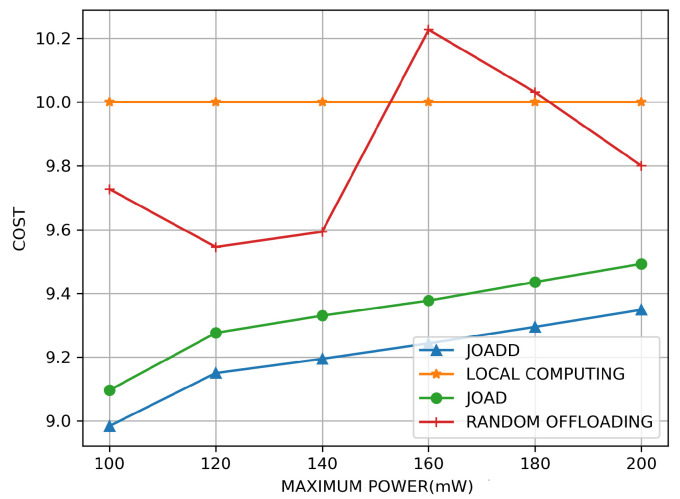
The effect on cost of different maximum transmission power.

Figure 6 illustrates the effect on cost under different maximum transmission power of VUE. As shown in this figure, the cost of local computing is a straight line on the top in orange. The reason for this phenomenon is that all tasks will be computed in the local MEC servers rather than being offloaded to the other MEC servers in the adjacent cells when the local computing model is selected. Hence, the cost of the local computing strategy is constant. Meanwhile, the cost of the random offloading strategy in this figure is displayed as a fluctuant polygonal line in red. That is because the random offloading strategy picks the offloading policy from all available policies randomly. When VUE obtains more computational resources that the MEC controller decided on, the MEC servers will speed up the computation; therefore, the cost reduces, and vice versa—the cost will rise. Finally, it is easy to observe that the cost of JOAD and that of JOADD grow with the increase of the maximum transmission power. Although the delay cost of task transmission decreases as the maximum transmission power increases, the total cost is linear with respect to the transmission power and more than the delay cost, which is logarithmic with respect to the power. Furthermore, due to eliminating the overestimation, JOADD is always better than JOAD.

**Figure 7:**
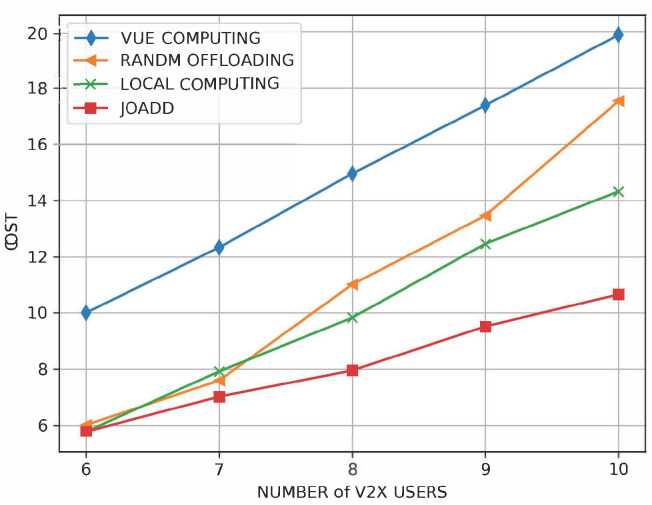
The effect on cost of the number of pieces of VUE.

Figure 7 shows the performances of different algorithms under different numbers of pieces of VUE. Obviously, the cost of all algorithms increases with the growth of VUE quantity. The cost of the VUE computation strategy is linear with respect to the number of pieces of VUE. The reason for the above phenomenon is that all pieces of VUE compute their tasks using constant local computational resources. Moreover, there is almost no difference between JOADD and JOAD when the number of pieces of VUE is small. In contrast, the difference rises as the number of pieces of VUE increases. Specifically, the cost of JOADD is about 30% less than that of the local computation strategy when the number of pieces of VUE equals 10. Even though the cost always rises, JOADD improves in performance significantly through balancing the load of MEC servers with the more efficient policy.

**Figure 8:**
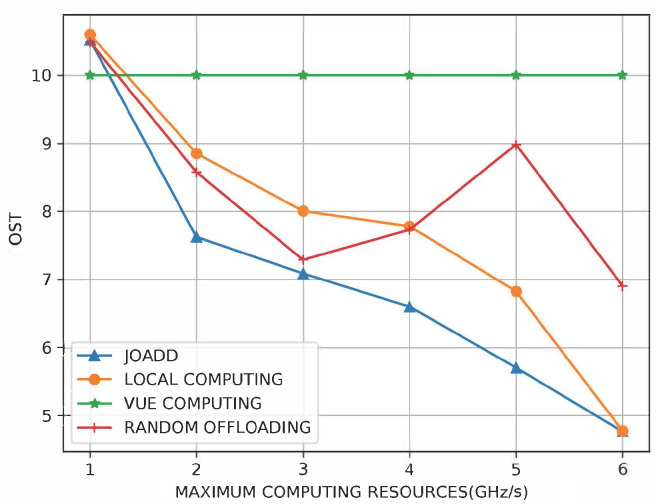
The effect on cost of the different maximum computational resources of the MEC server.

We studied the performance under different maximum computational resources of the MEC server in Figure 8. The performance of the VUE computation strategy is independent of the varying computational resources of MEC servers because the tasks are computed by themselves. Thus, the cost of it is constant. As a result of offloading tasks to MEC servers, the cost of JOADD and that of the local computation strategy decrease with the growth of the maximum computational resources of MEC servers. In addition, the cost of JOADD reduces faster than that of the other algorithms due to providing more flexible and better allocation policy. Although JOADD has an almost identical performance to the local computation strategy when the maximum computational resources of MEC servers are 1 and 6 GHz/s respectively, the reasons are different. When the maximum computational resources are equal to 1, the cost of JOADD and that of the local allocation strategy have little difference for the lesser amounts of computational resources. Moreover, the cost of JOADD and that of the local computation strategy are more than that of the VUE computation strategy due to the delay cost of the transmission. Besides, there is no obvious difference between the performance of JOADD and that of the local allocation strategy, because the computational resources are sufficient to satisfy the locally computing requirement. Specifically, the cost of JOADD is down to around 15% than that of the local computation strategy when the maximum computational resources equals 4 GHz/s.

**Figure 9:**
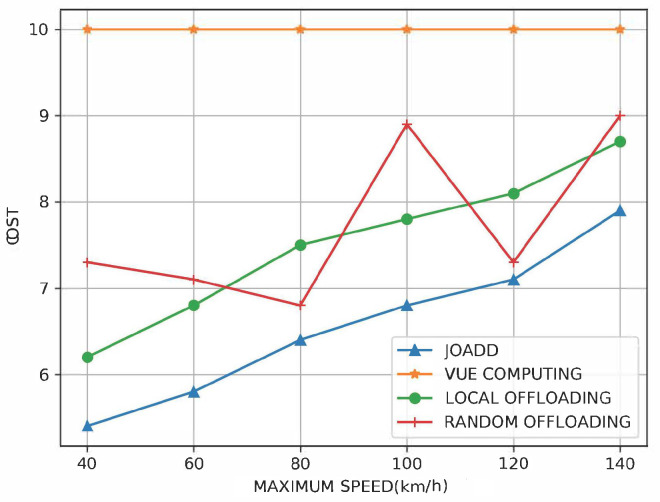
The effect on cost of the different speeds of vehicles.

Figure 9 shows the performance under the different vehicle speeds. Although the speed is not the variable that needs to be optimized, it can affect the channel gain according to (6). When the tasks are computed by VUE, no matter how fast the vehicles are, the cost always is a constant. However, the performance of all offloading strategy declines as the speed increases, no matter offloading to the local MEC server or other MEC servers. Moreover, when the speed of the vehicles reaches the normal speed (60–80 km/h), the cost of JOADD is less than that of the local offloading strategy by around 17%.

## 6. Conclusions and Future Work

In this paper, we studied a joint optimization problem in a multi-cell scenario with multiple pieces of VUE. This problem is one of the common NP-hard problems, which cannot be solved in the polynomial time. Consequently, we rewrote the origin object with the help of the concept of reinforcement learning. Then we proposed an algorithm based on double DQN to jointly optimize the decision variables, including the task offloading decision and the transmission power policy of VUE, and the computational resources allocation policy of MEC servers. Furthermore, to accelerate the speed of the training neural networks and reduce the latency of the algorithm, we cleaned the action space by discarding several inefficient actions. The simulation results demonstrate that our proposed algorithm outperforms algorithms based on other reinforcement learning or deep reinforcement learning methods. In the different scenarios, our proposes algorithm improved the performance in terms of convergence, defined cost, and speed by around 30%, 15%, and 17% compared with other algorithms, respectively. Our work is helpful for the applications of MEC in the V2X communications scenarios and breaking through the bottlenecks of cloud-based processing, i.e., long latency and massive data transmission.

Although task offloading decisions based on the dynamic vehicle environment are solved by our proposed algorithm basically, there are some limitations in our work to be left for future work. This paper considered a binary decision to decide whether to offload the whole task to MEC servers for calculation. However, the detailed offloading decisions, i.e., partial offloading decisions, are practical but more intricate, and will be left to future work. The optimization of the weights of delay and energy consumption will be left as our future work as well.

## Figures and Tables

**Table 1:** QoS requirements of advanced V2X applications supported by 5G-V2X.

Application Scenarios	Max End-to-End Latency (ms)	Reliability (%)	Data Rate (Mbps)
Vehicle Platooning	10–500	90–99.99	50–65
Advanced Driving	3–100	90–99.99	10–50
Extended Sensors	3–100	90–99.99	10–1000
Remote Driving	5	99.99	Uplink:25; Downlink 1

**Table 2:** Comparison of references of computation offloading (CO) and communication resources (CR). (*√*) indicates that the topic is covered. (×) indicates that the topic is not covered.

Year	Reference	Focus		Methods
		Computing Offloading (CO)	Communication Resources (CR)	
2015	[20]	*√*	*√*	Coalition Game
2015	[16]	*√*	×	Game Theory
2015	[17]	*√*	×	Potential Game
2017	[5]	*√*	×	Mixed Integer Linear Programming (MILP) model
2017	[10]	*√*	×	Convex Optimization Theory
2017	[21]	*√*	*√*	Evolutionary Game
2017	[19]	*√*	×	Queueing Networks Theory and Convex Optimization Theory
2019	[22]	*√*	×	Deep Reinforcement Learning (DRL)
2019	[6]	*√*	*√*	Game Theory and Convex Optimization Theory
2020	[23]	*√*	×	Convex Optimization Theory
2020	[18]	*√*	×	Graph Theory

**Table 3:** Parameters of communications.

Parameters	Value
*R*	500 m
$N_{0}$	−174 dBm/Hz
*B*	500 kHz
$t_{w}$	1
$f_{v}$	1 GHz/s
$d_{v}$	[500, 800] kbits
$c_{v}$	[900, 1100] Megacycles
*M*	3
*V*	6, 7, 8, 9, 10
*F*	1, 2, 3, 4, 5, 6 GHz/s
*P*	100, 120, 140, 160, 180, 200 mW
*V*	40, 60, 80, 120, 140 kM/h
$\beta_{1}^{l},\beta_{2}^{l},\beta_{1}^{u},\beta_{2}^{u}$	0.5

**Table 4:** The parameters of neural networks.

Parameters	Value
γ	0.1
α	0.09
ϵ	0.99
mini-batch	32
reply experience buffer’s size	500
optimizer	Adam
activity function	ReLu

## Data Availability

Data sharing not applicable.
